# The Complexity of the Arterial Blood Pressure Regulation during the Stress Test

**DOI:** 10.3390/diagnostics12051256

**Published:** 2022-05-18

**Authors:** Naseha Wafa Qammar, Ugnė Orinaitė, Vaiva Šiaučiūnaitė, Alfonsas Vainoras, Gintarė Šakalytė, Minvydas Ragulskis

**Affiliations:** 1 Department of Mathematical Modelling, Kaunas University of Technology, Studentu St. 50-146, LT-51368 Kaunas, Lithuania; naseha.qammar@ktu.edu (N.W.Q.); ugne.orinaite@ktu.lt (U.O.); vaiva.siauciunaite@ktu.lt (V.Š.); 2 Institute of Cardiology, Lithuanian University of Health Sciences, Sukileliu St. 17, LT-50161 Kaunas, Lithuania; alfonsas.vainoras@lsmuni.lt (A.V.); gintare.sakalyte@lsmuni.lt (G.Š.)

**Keywords:** arterial blood pressure, stress test, cardiac intercals

## Abstract

In this study, two categories of persons with normal and high ABP are subjected to the bicycle stress test (9 persons with normal ABP and 10 persons with high ABP). All persons are physically active men but not professional sportsmen. The mean and the standard deviation of age is 41.11 ± 10.21 years; height 178.88 ± 0.071 m; weight 80.53 ± 10.01 kg; body mass index 25.10 ± 2.06 kg/m${}^{2}$. Machine learning algorithms are employed to build a set of rules for the classification of the performance during the stress test. The heart rate, the JT interval, and the blood pressure readings are observed during the load and the recovery phases of the exercise. Although it is obvious that the two groups of persons will behave differently throughout the bicycle stress test, with this novel study, we are able to detect subtle variations in the rate at which these changes occur. This paper proves that these differences are measurable and substantial to detect subtle differences in the self-organization of the human cardiovascular system. It is shown that the data collected during the load phase of the stress test plays a more significant role than the data collected during the recovery phase. The data collected from the two groups of persons are approximated by Gaussian distribution. The introduced classification algorithm based on the statistical analysis and the triangle coordinate system helps to determine whether the reaction of the cardiovascular system of a new candidate is more pronounced by an increased heart rate or an increased blood pressure during the stress test. The developed approach produces valuable information about the self-organization of human cardiovascular system during a physical exercise.

## 1. Introduction

In a variety of studies, the human body is referred to as a dynamical adaptive system [1]. The human cardiovascular system is responsible for a variety of vital tasks, including the supply of blood and energy to the entire body. Cardiac regulation is a multi-level control system that acts both directly on a cardiac function and the whole circulatory functions in the human body. Rhythmic cardiac activity can be modulated directly through the cardiac energy system, through the function of receptor structures at various levels, through the functioning of autonomic nervous system chains or their interactions at the peripheral and central levels, and through the modulating effect of the central nervous system and the hormonal system [2,3]. The information on pressure, volume, and chemical-sensitive receptors in the cardiovascular system is integrated into the brainstem centers that coordinate the levels of blood pressure, vascular resistance, and cardiac systolic volume and its filling through the efferent nervous system and humoral connections. The level of regulation of the cardiovascular system enables the realization of blood redistribution through changes in blood flow and maintains a constant perfusion pressure. The regulation of the cardiovascular system is also performed through the limbic–hypothalamic system, which receives information from the above-mentioned levels of regulation. A distinction is made between long-term, intermediate-term, and short-term or rapid circulatory regulation mechanisms [2]. Rapid regulatory mechanisms play a key role in physical exercise, with sympathetic nerve-controlled vasodilatory vascular responses, baroreceptor reflexes, chemoreceptor reflexes, and CNS ischemia reflexes. A common feature of all these reflexes is a very rapid (within seconds) response to the oxygen circulation and energy demands of organs and tissues. Nerve regulation is complementary to hormonal regulation, including the need for adrenaline, noradrenaline, and a slightly longer latent vasopressin [2,3,4]. The predominant cardiovascular regulatory mechanisms and their interactions can be described if the conditions of exercise are precisely defined [5]. From the very beginning of exercise, the heart rate often increases immediately. This is due to a very rapid autonomic response of the nervous system to exercise as well as to the termination of the influence of the vagus nerve [6]. The frequency of the heart rate is proportional to the intensity of exercise [7]. At the high intensity of exercise, the heart rate increases due to an increase in sympathetic influence [3,8]. The heart rate rises during exercise to satisfy the demands of working muscles. It is known that the heart rate decreases exponentially in many cases immediately after exercise. Parasympathetic inhibition and blood catecholamine levels are thought to recover after exercise with decreased exposure to the upper nerve centers and peripheral nerves [9]. The CNS acts mainly on the heart through the extracardiac nerves of the autonomic nervous system and the humoral pathway [8]. It should be noted that the effect of the autonomic nervous system on heart rate regulation is much faster than the humoral one. An important functional indicator of cardiac activity is the arterial blood pressure (ABP). ABP increases in proportion to exercise intensity and active muscle mass [10]. As the minute volume and the heart rate (HR) are increased, the *n. vagus* influence is decreased, and ABP begins to rise because of the sympathetic activation [11]. If small muscle groups are involved during the exercise, then an increase in the sympathetic nerve activation does not cause vasoconstriction. If large muscle groups are involved in a physical exercise, sympathetic muscle activation plays an important role in maintaining ABP for muscles working through blood flow regulation in upper rather than lower limb muscles [12]. Such an ABP response is associated with the fact that the muscles of the upper extremities are smaller, and this leads to greater peripheral resistance than in the large muscles of the legs, which reduces their blood circulation more than at the beginning of the exercise. This is due to the activation of α-adrenoceptors and an increase in catecholamine levels [13]. The amount of catecholamines increases with the intensity of exercise; it is low with low exercise. Blood pressure response to physical exercise is a systemic, significant diagnostic parameter measured in diagnostic laboratories during submaximal physical exercise tests. The increase in the workload is expected to raise systolic blood pressure during complex, isotonic exercises. The systolic arterial blood pressure rises during a dynamic exercise in clinically stable patients and stabilizes after 2–3 min of the exercise at a given intensity [14,15]. In such cases, the diastolic blood pressure usually remains constant or decreases slightly [14]. European and American experts state that (in normal conditions), high-intensity dynamic exercise raises systolic blood pressure to 250 mmHg and diastolic blood pressure to 110 mmHg [16,17]. According to the American College of Sports Medicine, increasing the intensity of the dynamic exercise by 1 MET can result in a 10 mmHg increase in systolic blood pressure [18]. Stair-type response to exercise is characterized by a stepwise rise in systolic ABP after the second and the third minutes of the recovery of the exercise. The recovery of ABP and HR is protracted. This indicates insufficient blood flow regulation and a functional decrease in the cardiovascular system’s capacity [19]. It is shown that the excessive increase of the blood pressure during an exercise test is a prognostic factor for potential hypertension or mortality due to cardiovascular problems [20]. The processes under consideration are defined by parameters that do not provide the physician with a specific physiological meaning. As a result, the use of those methodologies based on stress tests is rather limited for diagnostic purposes. It is still relevant to develop and use a simple, physiologically based model described by basic cardiac parameters, taking into account the normative values of the parameters (and their variation) in order to develop a suitable model for diagnostic purposes. Nonlinear analysis methods of functional state analysis are still rarely used in practice for several reasons. Such an analysis is one of the recent methodologies used in the practice of clinical medicine science [21]. Several statistical methodologies are used to assess the distribution of a dataset at various functional levels in clinical and research contexts. Mathematical and computational models can be used to standardize data integration by providing a comprehensive picture of the data as well as the ability to compute critical metrics [22]. Machine learning techniques are used to predict cardiac diseases based on the Gaussian distribution fitted to the extracted features of clinical data in [23]. The distributions of echocardiographic values of ventricular diameter in relation to age, gender, body size, and race using the Gaussian distribution is exploited for the diagnostic purposes in [24].

In recent years, several techniques have been proposed for the identification of hypertension at an early stage. Echocardiographic markers of pulmonary hemodynamics and right ventricular hypertrophy in rat models of pulmonary hypertension are developed in [25]. The automated detection of severity of hypertension from ECG signals using an optimal bi-orthogonal wavelet filter bank is proposed in [26]. An automated diagnostic tool for hypertension using convolutional neural network is presented in [27]. A two-stage deep CNN architecture for the classification of low-risk and high-risk hypertension classes using multi-lead ECG signals is reported in [28]. ECG signal-based automated hypertension detection using the Fourier decomposition method and cosine-modulated filter banks is described in [29]. A review of the assessment techniques for hypertension based on clinical electrocardiogram features is given in [29]. Recent advances in deep learning-based classification frameworks are discussed in [30,31].

The reaction of the human cardiovascular system during physical exercise is usually represented in two basic ways: one is the increased heart rate, and the other is the increase in the blood pressure. This occurs for every individual, but the significant element worthy of consideration is the rate at which these changes occur within a human body during the exercise. In this novel study, we will examine how the human body reacts to physical exercise and how the regulatory system controls the processes occurring during the stress test. It is well known that persons with high blood pressure behave differently during the bicycle stress test than those who do not have the high ABP. We hypothesize that while both categories of persons (one with normal ABP and other with high ABP) experience an increase in the heart rate and the blood pressure, there are subtle differences in the rate at which these changes occur. In this paper, we will show that these subtle differences are measurable and significant, and that after studying cohorts of persons with increased ABP and those with normal ABP, an accurate categorization of a new person can be made by means of machine learning algorithms. We will further demonstrate that this classification can be performed using statistical features identified only from the load but not the recovery data. We will also demonstrate and visualize how the regulating system governs the processes that occur within the cardiovascular system during the exercise.

### The Model of Integral Evaluation of the Human Cardiovascular System

**Figure 1:**
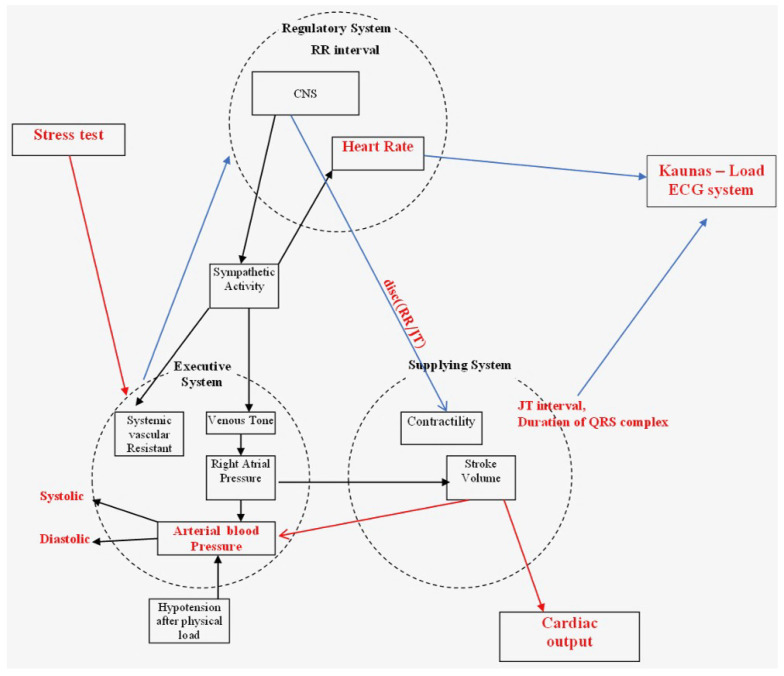
A schematic diagram illustrating the interconnectivity of the Executive System, the Supply System, and the Regulatory System.

The model of integral evaluation of the human cardiovascular system is presented in Figure 1. This model is inspired by a recent study [32], and it is also adapted [33] for the study of complex processes in the cardiovascular system during electrical auricular vagus nerve stimulation. This model singles out three basic parts: the Regulatory System, the Supply System, and the Executive System. As mentioned previously, the stress test stimulates the sympathetic nervous system. To accommodate the increased metabolic activity in the skeletal muscles, the circulatory system must properly control the transport of oxygen and carbon dioxide as well as help to buffer the pH level of active tissues. The amount of blood pumped by the left ventricle per minute (the cardiac output) is expressed as liters/minute:

(1) Q=(HR)·(SV),

where *SV* is the stroke volume. With a stepping working rate, the cardiac output increases in a nearly linear fashion to meet the increased oxygen demand, and its output can be measured by echocardiography. The consumption of oxygen $VO_{2}$ can be expressed by the Fick equation. This equation is expressed as:

(2) $$VO_{2}=\left(CO\right)\cdot \left(DAVO\right),$$

where *CO* is cardiac output, *DAVO* is the difference in arterial and venous oxygen levels, and $VO_{2}max$ is a measure of the aerobic exercise capacity and is defined as the highest rate of oxygen uptake an individual can maintain during intense activity [34]. This is accomplished by increasing cardiac output (increased heart rate and stroke volume) and the modulation of microvascular circulation. In addition, the action of local vasodilators such as nitric oxide from endothelial cells helps to ensure adequate blood flow.

In addition, systolic blood pressure rises linearly to peak values of 200 to 249 mmHg for normotensive individuals, while diastolic blood pressure remains near the resting levels. Hypertensive people have higher systolic blood pressures at a given rate of work, and they can also have higher diastolic blood pressures. The peripheral resistance to blood flow is proportional to the vessel’s diameter and length as well as blood viscosity in the peripheral vessels [35]. Under physical stress, the vessels dilate, increasing their diameters. Hypertension patients have increased peripheral resistance compared to the general population, which is a major cause of their higher average blood pressure. Blood pressure falls below pre-exercise levels two to three hours after exercise, which is a condition known as “post-exercise hypotension”. Figure 1 depicts the schematic diagram of the regulatory system, the executive system, and the supply system. When the exercise lasts more than a few seconds, it is critical to provide the skeletal muscles with an adequate amount of oxygen and energy for the execution of the load. Adequate provision of the movement and the support systems during the exercise is possible when the supply systems are activated, which is ensured by the regulatory systems [36,37]. As a result, when assessing the body’s response to the physical activity, the three systems must be considered: the executive system (functioning muscles); the regulatory system (including the central nervous system (CNS), autonomous, and humoral management systems); and the supply system (the cardiovascular system) [38]. This model describes the performance of all three systems during the exercise. The changes during the exercise involve the mechanism of hemodynamic redistribution, the main purpose of which is to adequately adapt the body to perform the required load to assist the body’s basic systems in adapting to the load.

**Figure 2:**
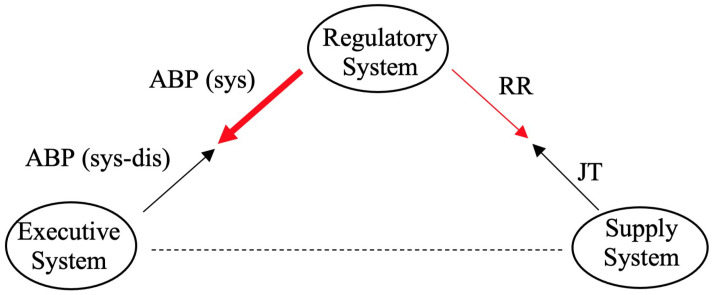
A simplified schematic diagram illustrating the interconnectivity of the Executive System, the Supply System, and the Regulatory System.

When an organism reaches a certain capacity during the exercise, complex relationships between the three systems (Figure 2) are activated [33]. The following main elements in Figure 2 can be distinguished based on the human physiology, which determine the main changes in the body during exercise: (1) The Regulatory System is in charge of regulating the various processes in the human body; (2) The Executive System is in charge of active, working muscles; and (3) The Supply System is in charge of ensuring adequate hemodynamics. The three systems are interconnected in Figure 2, with the systolic ABP (sys) representing a link from the Regulatory System to the Executive System and the difference in systolic and diastolic blood pressures, i.e., the ABP (sys-dis), representing a link from the Executive System to the Regulatory System (this branch of the triangle is responsible for the blood pressure rate).

The RR and JT cardiac intervals represent the relationship between the Regulatory System and the Supply System on the right side of the triangle in Figure 2. This branch represents the variation of the heart rate. A single ECG characteristic, such as the RR interval, which is a systemic parameter, reflects the functional state of the total organism’s regulatory system, and it is regarded as a measure of neurocardiac function that reflects the heart–brain interaction and the dynamics of the autonomic nervous system (ANS) [39,40]. RR variation interval reflects the inherent self-regulatory capacity, adaptability, and resilience of the cardiovascular system [40,41].

The JT cardiac interval reflects the duration of the ventricular repolarization. JT dispersion during the stress test reflects the heterogeneity of myocardial repolarization duration and its variation [42]. It is associated with local myocardial ischemia and cardiac wall movement disorder [43,44]. The JT interval encompasses different electrophysiological phenomena and is divided into the JTa interval (from point J to the vertex T) and the Te interval (from the peak of the T wave to the end of T). Changes in the JT interval are influenced by the regulatory nervous system. Metabolic changes in the body are associated with changes in repolarization. Derivatives with a shorter JT interval indicate earlier repolarization in the reflected myocardial zones, faster metabolic changes, and where the longer JT indicates slower repolarization, slower metabolic responses. This phenomenon is known as JT interval variance (JTd), which indicates myocardial inhomogeneity and electrical instability. The JT interval shortens to 160 ms during the exercise in healthy individuals [38,44]. Meanwhile, in patients with ischemic heart disease, the JT interval is significantly shorter during the exercise.

It should be noted that in Figure 2, we are not focusing on the relationship between the Supply System and the Executive System, so it is represented by a dashed line. The primary goal of this research is to achieve a delicate balance between the two red lines in Figure 2. As previously stated, each individual influenced by the load will experience an increase in the heart rate and the blood pressure. However, the primary goal is to identify a fine balance between these two.

Figure 2 shows a thin arrow on the right side of the triangle and a thick arrow on the left side of the triangle to indicate that this applies to a person whose response to a load is resulting into a more increased blood pressure than into an increased heart rate. However, if the diagram was arranged with a thick right arrow and a thin left arrow, we would infer that it relates to a person whose regulatory response to the load is more characterized by an elevated heart rate than by a blood pressure.

## 2. Methods

### 2.1. The Description of the Experimental Setup

The current study fulfilled all the standards for experimentation ethics. The approval to perform biomedical investigations was provided by the Kaunas Regional Ethics Committee for Biomedical Investigations, No. BE-2-51, on 23 December 2015. An ECG stress test on a cycle ergometer was used to record cardiac intervals and their parameters. The “Kaunas-Load” system developed at the Institute of Cardiology (Lithuanian University of Health Sciences) is used to perform the synchronous registration of 12 leads and different standard ECG parameters (including the duration of JT and the amplitude of ST intervals for each cardiac cycle) [45,46].

The ECG is registered at the start of the bicycle ergometry exercise with the load set to 50 W. The load is held constant for two minutes before being increased by 50 W. Throughout the bicycle ergometry exercise, the person is required to maintain a constant cycle ergometer spinning rate of 60 revolutions per minute. The stress test is terminated when the person fails to maintain this spinning rate, or first clinical indications for load limitation are observed according to the AHA (American Heart Association).

The cohort comprised 19 physically active men but not professional sportsmen. The mean and standard deviation of age is 41.11 ± 10.21 years; height 178.88 ± 0.071 m; weight 80.53 ± 10.01 kg; body mass index 25.10 ± 2.06 kg/m${}^{2}$.

### 2.2. The Algorithm for the Identification of the RR/JT Algebraic Relationship

The RR and JT cardiac intervals are continuously and synchronously recorded during the load and recovery processes and denoted as time series $x=(x_{1},x_{2},\ldots ,x_{n})$ and $y=(y_{1},y_{2},\ldots ,y_{n})$; where *n* is the number of recorded heart beats during the whole bicycle ergometry exercise. We will use the algorithm for the reconstruction of the algebraic relationships between two time series *x* and *y* as introduced in [47]. All computational operations can be grouped into four basic steps.

**Step # 1**. Let us fix the current discrete time moment (denoted as *k*) and time lag $\delta \in 1,2,\ldots \delta_{max}$, where $\delta_{max}$ is the upper bound for the time lag. The six elements of both time series $x_{k-\delta },x_{k},x_{k+\delta },y_{k-\delta },y_{k},y_{k+\delta }$ (current, backward and forward time lagged measurements) are mapped into a two-dimensional perfect matrix of Lagrange differences [47]. A set of the following requirements is raised for perfect matrices of Lagrange differences [47]: (a) all elements of the matrix must be different; (b) zeroth-order differences are located on the main diagonal; (c) first-order differences are located on the secondary diagonal; (d) the matrix is lexicographically balanced (the number of elements from the first and the second time is the same); (e) the matrix is balanced in respect of time (the sum of all time lags is equal to zero). The number of different perfect matrices of Lagrange differences is 18 [47]. For example, the first perfect matrix of Lagrange differences $L_{\delta ,k}^{\left(1\right)}=\left[\begin{array}{cc} x_{k} & x_{k+\delta }-y_{k+\delta }\\ x_{k-\delta }-y_{k-\delta } & y_{k} \end{array}\right]$ is used in [48]. Two time series are mapped into a sequence of trajectory matrices $L_{\delta ,k}^{\left(\beta \right)};k=\left(1+\delta ,2+\delta ,\ldots ,n-\delta \right);\beta \in 1,2,\ldots ,18$.

**Step # 2**. The sequence of matrices $L_{\delta ,k}^{\left(\beta \right)}$ is transformed into a single scalar time series using a mapping $F:R^{\left(2\times 2\right)}\to R^{1}$. The mapping F can be defined in different ways. For example, the maximal modulus of the two eigenvalues of the matrix $L_{\delta ,k}^{\left(\beta \right)}$ is used in [47]; the norm of the matrix $L_{\delta ,k}^{\left(\beta \right)}$ is used in [48]. In this paper, we define the mapping F as the discriminant of the matrix $L_{\delta ,k}^{\left(\beta \right)}:F\left(L_{\delta ,k}^{\left(\beta \right)}\right)=disc\left(L_{\delta ,k}^{\left(\beta \right)}\right)={\left(\alpha_{11}-\alpha_{22}\right)}^{2}+4\alpha_{12}\alpha_{21}$, where indexes denote the coordinates of elements in the matrix $L_{\delta ,k}^{\left(\beta \right)}$. The discriminant of the matrix is chosen instead of another mappings used in [47,48] because the value of the discriminant of the perfect matrix of Lagrange differences tends to zero when the variability of time series *x* and *y* becomes similar [47]. This feature helps to bring the RR/JT algebraic relationship almost down to zero when the collapse of complexity happens at the end of the load phase of the stress test [47].

**Step # 3**. In this step, the internal and external smoothing is applied for the scalar sequence $F(L_{\delta ,k}^{\left(\beta \right)})$. If the radius of the internal smoothing is denoted by $R_{i}$ and the radius of the external smoothing is denoted by $R_{e}$, then the smoothed sequence depicting the algebraic relationship between the two time series *x* and *y* reads:

(3) $$s_{k}\left(R_{i},R_{e},\beta \right)=\frac{1}{R_{i}\left(2R_{e}+1\right)}\sum_{j=k-R_{e}}^{k+R_{e}}\sum_{\delta =1}^{R_{i}}i=F\left(L_{\delta ,k}^{\left(\beta \right)}\right)$$

where $k=\left(1+R_{i}+R_{e}\right),\left(2+R_{i}+R_{e}\right),\ldots ,\left(n-R_{i}-R_{e}\right)$. A well-posed optimization problem in respect of the smoothing parameters is formulated in [47] for the whole cohort of persons resulting in $R_{i}=3$, $R_{e}=4$ and β=1. These values of the parameters are kept fixed in this paper, too. All further numerical computations are based on $s_{k}\left(3,4,1\right)$.

**Step # 4**. After establishing the algebraic relationships, the statistical operations are performed for the cohort. First, the algebraic relationship between the RR and JT interval is computed for each person and averaged for each minute during the exercise. Then, the arterial blood pressure data (once per minute) are measured during the exercise. Following that, the dataset is put to the linear regression for each individual person. The Gaussian distribution of the slope coefficients of the linear regression computed for each person in the cohort is then applied to the whole dataset. Finally, the one sigma rule is used to perform statistically meaningful difference between the two cohorts (persons with normal ABP and persons with high ABP) to perform the classification. All these steps are discussed in detail below.

### 2.3. The Structure of Machine Learning Algorithms for the Classification

The structure of machine learning algorithms used for the classification of the performance during the stress test is presented in this section. The extracted knowledge used for the classification is based on the slope coefficient of the regression between the averaged RR/JT algebraic relationship and ABP data in the phase plane. The extracted data for two cohorts is fitted into two separate Gaussian distributions. The variance interval used for the classification is based on the one sigma rule for the two distribution functions. Detailed explanations for the construction of machine learning algorithms is presented in the following subsections.

#### 2.3.1. The Averaged RR/JT Algebraic Relationship and ABP Data in a Phase Plane

**Figure 3:**
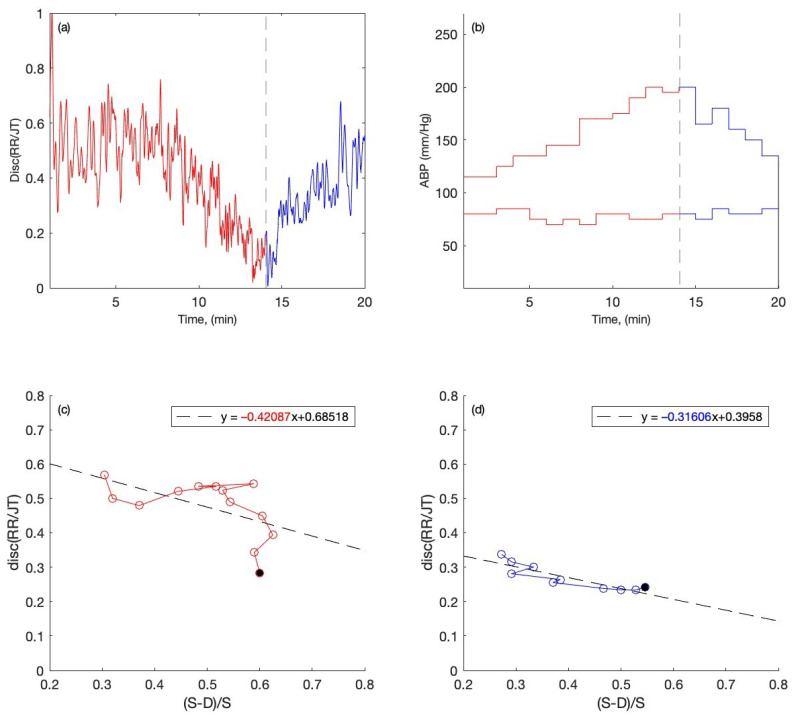
The readings are presented for the person with normal ABP. (**a**) Disc (RR/JT) changes up to 14 min during the load and after 14 min during recovery. (**b**) Variations in Systolic and Diastolic ABP during the stress test. (**c**) The phase plane for the parameters X = (S − D)/S and Y = Disc (RR/JT) and the linear regression plot, where the data for each parameter are presented as a one-minute mean during the load and (**d**) during the recovery.

**Figure 4:**
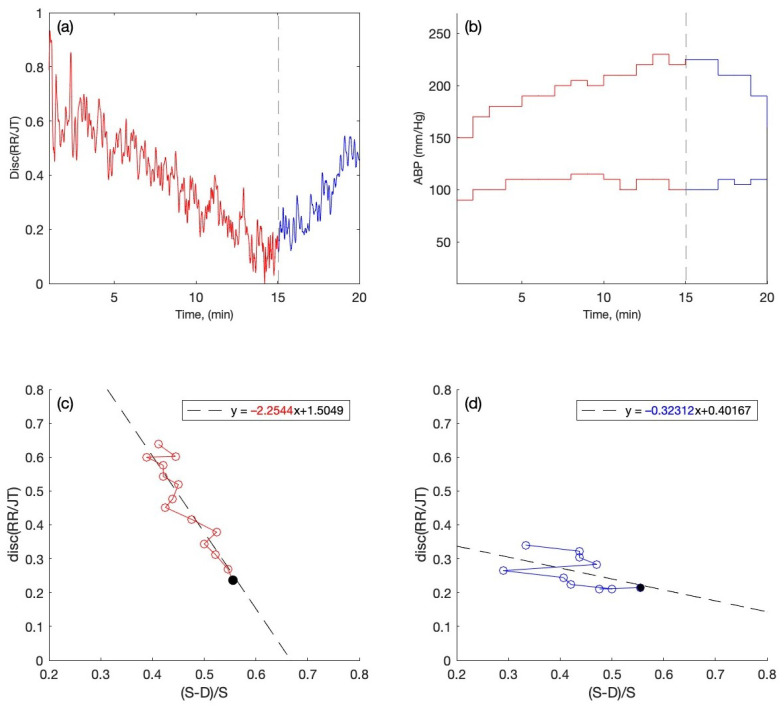
The readings are presented for the person with high ABP. (**a**) Disc (RR/JT) changes up to 15 min during the load and after 15 min during recovery. (**b**) Variations in Systolic and Diastolic ABP during the stress test. (**c**) The phase plane for the parameters X = (S − D)/S and Y = Disc (RR/JT) and the linear regression plot, where the data for each parameter are presented as a one-minute mean during the load and (**d**) during the recovery.

Overall, nineteen people were tested: 9 of them had normal ABP and 10 had high ABP. In Figure 3a and Figure 4a, the algebraic relationships for the cohort of individuals are computed and averaged for each minute for one person with normal ABP and one person with high ABP during the stress test. The red graph shows the averaged algebraic relationship during the load phase of the stress test. The black dashed line represents the end of the load phase and beginning of the recovery phase during the stress test. The blue graph is the visualization of the recovery phase data, which are recorded while the individual is performing the stress test. The *x*-axis represents a one-time unit (in minutes), while the *y*-axis represents the data recordings of cardiac intervals (discriminant RR/JT).

Figure 3b and Figure 4b show the systolic and diastolic blood pressure readings during the stress test for one person with the normal ABP and one person with the high ABP. The *x*-axis represents the time scale in minutes, while the *y*-axis represents the systolic and diastolic ABP readings in millimeters of mercury (mmHg). The red graphs show the ABP data collected during the stress test when the person’s body has been under load. The black dashed vertical line indicates the termination of the load phase and the start of the recovery phase. The blue graph depicts the ABP reading during the stress test’s recovery phase.

In Figure 3c,d and Figure 4c,d, the phase plane plots are constructed for the persons with normal and high ABP during the stress test with the recordings of both load and recovery phase. The phase plane plots are constructed with the data obtained from the systolic and diastolic blood pressure data (S − D)/S (per minute) on the *x*-axis and the averaged algebraic relationship (RR/JT) on the *y*-axis. This helps to perform the linear regression for the cohorts of individuals. From the linear regression, the slope coefficients for the individuals during the load and recovery phase are obtained for further statistical analysis. For instance, in Figure 3c and Figure 4c, the slope coefficients for the person with normal ABP during the load phase of the exercise are obtained after the linear regression, and the value of the slope coefficient is shown in red (−0.42087) and (−2.2544). Similarly, the slope coefficients obtained from the linear regression during the recovery phase of the exercise are shown in Figure 3d and Figure 4d in blue numerals.

As explained previously, the phase plane plots constructed with the data obtained from the systolic and diastolic blood pressure data (S − D)/S (per minute) on the *x*-axis and the averaged algebraic relationship (RR/JT) on the *y*-axis are now subject to linear regression. The application of linear regression helps with obtaining the slope coefficients for the load and recovery phases of the exercise for the cohort of individuals. Table 1 and Table 2 show the values of the slope coefficients obtained for all individuals during the load and recovery phase.

#### 2.3.2. Data Fitting into the Gaussian Distribution

After obtaining the slope coefficients for the cohort of persons throughout the load and recovery phases of exercise, the tabulated data in Table 1 and Table 2 are fitted to the Gaussian distribution to generate the baseline data for evaluating the new candidate slope coefficient within the variation interval. Once the distribution is confirmed to be really Gaussian, the variation interval is calculated. To confirm the normal distribution of the slope coefficients, the Anderson–Darling goodness-of-fit hypothesis test is performed. Following the test, the significant level obtained for both cohorts of persons throughout the load and recovery phases is tabulated in Table 3, which also confirms the normal distribution of the data.

#### 2.3.3. Classification Using the One Sigma Rule

**Figure 5:**
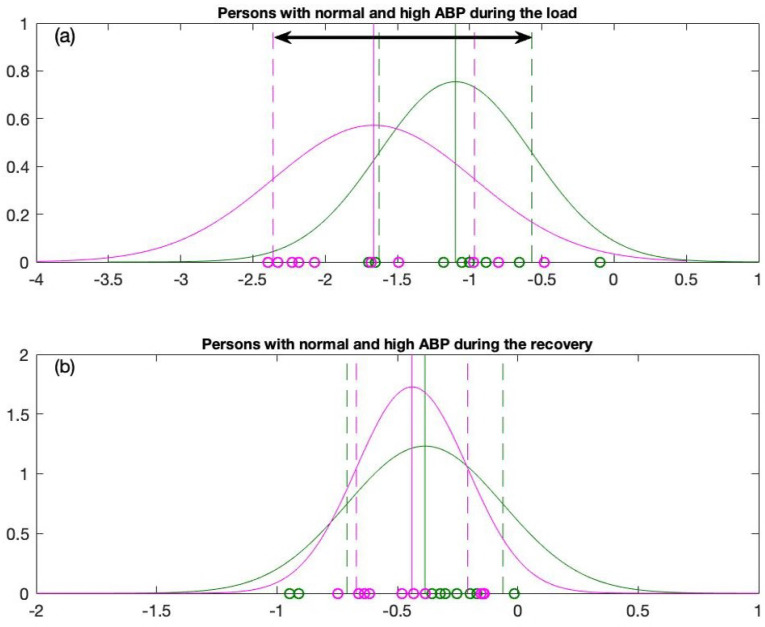
Overall comparison of normal distribution graphs for (**a**) Persons with normal and high ABP during the load and (**b**) Persons with normal and high ABP during the recovery.

The final step in the statistical analysis is to apply the one sigma rule to the baseline data acquired from the Gaussian distribution to the slope coefficients obtained during exercise for individuals with normal and high ABP. The application of the one sigma rule, $\mu_{L2}-\sigma_{L2}$ and $\mu_{L1}+\sigma_{L1}$ generates a variation interval (see Figure 5a). This variation interval will serve as the classification’s foundation and will aid in the evaluation of the new candidate slope coefficient behavior entering this interval. From a statistical standpoint, the classification should be meaningful under the constraints $|\mu_{L2}-\sigma_{L1}|\geq min\left[\left(\sigma_{L1},\sigma_{L2}\right)\right]$ for the load data and $|\sigma_{R2}-\mu_{R1}|\geq min[\left(\sigma_{R1},\sigma_{R2}\right]$ for the recovery data; see Table 3.

When the statistical data are collected and the distributions are in place, we may demonstrate the efficiency and effectiveness of this classification by introducing new candidates to test the functionality of the proposed algorithm. A coordinate system (see Table 4) that conforms to the integral evaluation model (see Figure 2) is also designed, in which the new candidates’ body response to exercise, particularly during the load process is visualized, and it can be seen which side of the triangle system the individual falls on, either toward the increased heart rate side or the increased blood pressure rate. The findings will be presented and discussed in depth in the section that follows.

## 3. Results and Discussions

First, we look at how the heart rate and systolic and diastolic blood pressure readings vary over time throughout the load and recovery phases of a bicycle stress test in one person with normal ABP and one person with high ABP. The plots in red color depict the data during the load process, while the plots in blue depict the data during the recovery process, and the black dashed line indicates the end of the load process and the beginning of the recovery process. Figure 3 represents the case of an individual with normal ABP in the first plot; Figure 3a shows the variation in Disc RR/JT vs. time (in minutes). As we can see, there is an increase in Disc RR/JT in the first few minutes of activity, which is quite typical for a person with normal ABP. This could indicate that the person’s body is attempting to restructure its condition in order to find the optimal amount of physiological function organization while under load. In the case of small loads, this is a common reaction for a person with normal ABP. Later, as the load increases, the Disc RR/JT decreases until it reaches a minimal value at the maximum load, indicating a maximal decrease in complexity, when the main task of the organism is to concentrate all systems’ potential to develop maximal muscle function and power to achieve the goal. When the load is removed, we see the complex restoration of Disc RR/JT to its original state. Figure 3b depicts the systolic and diastolic pressure readings during a bicycle stress test. Because the individual has normal ABP, his blood pressure measurements are very normal throughout the duration of the exercise. Figure 3c,d show the phase plane representation of the arterial blood pressure’s relative pulse amplitude during the load and recovery process, with (S − D)/S on the *x*-axis and Disc RR/JT on the *y*-axis. A full filled circle on the phase plane graph indicates the end of the load and beginning of the recovery process during the bicycle stress test. The linear regression built throughout the load and recovery phases helps us compute the slope coefficients for this specific person, which are −0.42087 for load and −0.31606 for recovery. It is also worth noting that the trend line coefficient values make sense, as the decrease in Disc RR/JT occurs at the same rate as the increase in relative pulse amplitude (S − D)/S.

Figure 4a depicts the fluctuation in Disc (RR/JT) vs. time (in minutes) during the load and recovery process in an individual with high ABP. There is no early rise in Disc (RR/JT) as there would be in a person with normal ABP (Figure 3a). This indicates that the individual’s body is not attempting to rearrange its condition in order to find the optimal degree of bodily function organization during load, since the person is hypertensive. Figure 4b depicts the variations in systolic and diastolic ABP during the experiment during each minute of load and recovery in a hypertensive person. The phase plane representation of the arterial blood pressure’s relative pulse amplitude of (S − D)/S on the *x*-axis and Disc (RR/JT) on the *y*-axis throughout the load and recovery process is illustrated in Figure 4c,d. We observe the slope coefficients during the load and recovery process, which are “−2.2544” during the load and “−0.32312” during the recovery.

**Figure 6:**
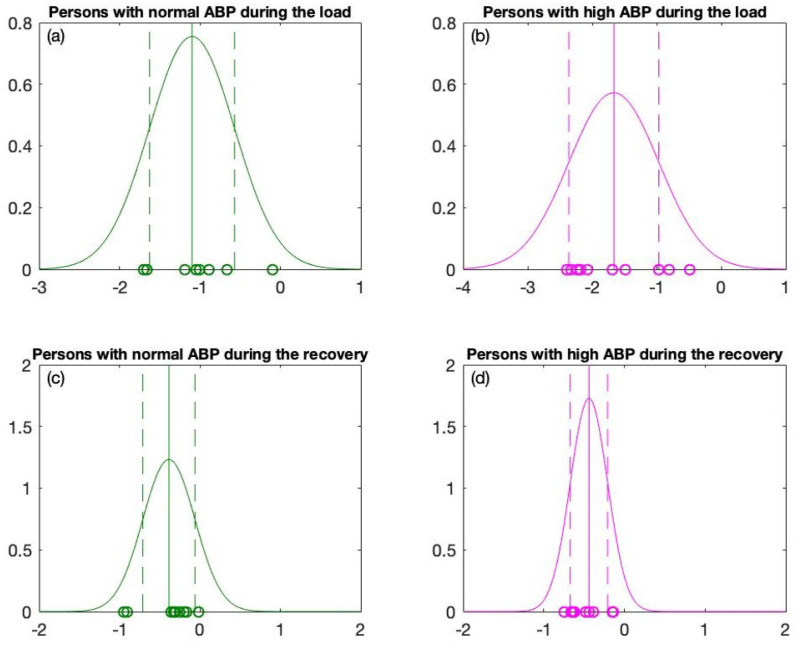
The Gaussian distribution graphs for (**a**) Persons with normal ABP during the load. (**b**) Persons with high ABP during the load. (**c**) Persons with normal ABP during the recovery and (**d**) Persons with high ABP during the recovery.

The case of one person with normal ABP (115-80 mmHg at the beginning of the stress test) and one person with high ABP (150–90 mmHg at the beginning of the stress test) is already discussed in detail in Figure 3 and Figure 4. Both individuals experience an increase in the heart rate and an increase in the blood pressure during the exercise. The person with high ABP behaves differently than the one who has normal ABP (Figure 3 and Figure 4). However, after applying the linear regression to the phase plane plots and getting the slope coefficient values, it is possible to observe that the slope coefficients for each individual are different. The slope coefficients reconstructed for each individual are grouped into four distinct sets according to the normal or high ABP and according to the load and the recovery data (see Table 1 and Table 2). Now, the Gaussian distribution is fitted to each separate set (see Figure 6). Next, to cross-validate the hypothesis that the slope coefficients are normally distributed according to the Gaussian distribution, we run the Anderson–Darling goodness-of-fit test [49]. The normal distribution of four data sets is under the significance levels indicated in Table 3.

Next, the one sigma rule is applied to the distribution graphs for the load and the recovery data (the distribution of slope coefficients) in order to design a statistically meaningful classification algorithm between persons with high ABP and normal ABP. The data for persons with normal ABP are plotted in green; the data for persons with high ABP are plotted in magenta (Figure 5 and Figure 6). The means of the reconstructed Gaussian distributions are plotted with solid vertical lines. As mentioned previously, we will use one sigma rule. Therefore, the left end $\mu_{L2}-\sigma_{L2}$ and right end $\mu_{L1}+\sigma_{L1}$ of the variation interval are plotted in vertical dashed lines (see Figure 6a,b). One sigma rule is likewise applied to the recovery data with left and right ends of the variation interval equal to $\mu_{R2}-\sigma_{R2}$ and $\mu_{R1}+\sigma_{R1}$ accordingly (see Figure 6c,d).

According to our observations from the distribution graphs, the variation interval during the load process is much longer compared to the variation interval reconstructed from the recovery data. In addition, the difference between the means (for persons with high ABP and persons with normal ABP) is much larger for the load data $\left(|\mu_{L2}-\mu_{L1}|=0.5654\right)$ than the recovery data $\left(|\mu_{R2}-\mu_{R1}|=0.0536\right)$. The statistical condition $|\mu_{L2}-\mu_{L1}|\geq min\left[\left(\sigma_{L1},\sigma_{L2}\right)\right]$ holds true only for the load data (see Table 3). On the contrary, this condition does not hold true for the recovery data $|\mu_{R2}-\mu_{R1}|\geq min\left[\left(\sigma_{R1},\sigma_{R2}\right)\right]$ (see Table 3).

The statistical indicators (Table 3) and single sigma variation intervals (Figure 5) demonstrate that the load data play the primary role in establishing subtle differences in the self-organization of the cardiovascular system during the stress test (as compared to the recovery data). The variation interval applicable for the classification of a new candidate based on his slope coefficient in the load data is denoted by the black horizontal arrow linking points $\mu_{L2}-\sigma_{L2}$ and $\mu_{L1}+\sigma_{L1}$ in Figure 5a. This variation interval will be our primary focus, as we will analyze new candidates in order to determine their cardiovascular response to the stress test during the load. Moreover, such a classification will help to determine the type of the response—whether this self-organization of their cardiovascular system is closer to an average person with high ABP or to an average person with normal ABP. Finally, this classification will be visualized in the form of the triangle system used to depict the interacting elements of the organism (Figure 2). From a statistical standpoint, the differences between the cohorts of high and normal ABP are clearly expressed only during the load. Therefore, from this point onwards, we will examine the response of the cardiovascular system only during the load process.

### 3.1. The Implementation of the Classification Model

A non-dimensional coordinate system is constructed for the visualization of the integral evaluation model depicted in Figure 2. The purpose of such a visualization is to represent the behavior of a new person according to the location of his slope coefficients in the variation interval proposed in this study (see Figure 5a). The branches linking the Regulatory System with the Executive System and the Regulatory System with the Supply System are plotted in lines of different thickness. The relative thickness of the lines corresponds to the location of the identified slope coefficient in the variation interval.

The three coordinates that correspond to the three systems in the triangle system are set to (−1,0), (0,1), and (1,0) (see Table 4). The left branch of the triangle represents the interaction between the Regulatory System and the Executive system. The right branch of the triangle represents the interaction between the Regulatory System and the Supply system. The left branch is plotted in a line of a maximal thickness if the slope coefficient is located at the left end of the variation interval. Analogously, the right branch is plotted in a line of a maximal thickness if the slope coefficient is located at the right end of the variation interval. In other words, the extracted knowledge from the cohort data is used to classify the performance of a new person during the stress test.

The mathematical classification of the person’s behavior during the stress test is based on a linear approximation inside the variation interval with cut outliers. The formula describing the classification is presented in Table 5. The slope coefficient of a new person is denoted as New, and the interpolation coefficient is denoted as C (Table 5). The maximal thickness of lines used to represent the left and the right branches is set to 10 pixels (the minimum thickness is 1 pixel). The interpolation coefficient C is computed according to one of the three conditions explicitly explained in Table 5. If the slope coefficient value of the new coming candidate is less than or equal to the left branch $\mu_{L2}-\sigma_{L2}$ of the variation interval, then interpolation coefficient (C) will be equal to negative one, and the thickness of the left branch of the triangle system will be according to the formula $C=10-\frac{C+1}{2}\cdot 9$, as shown in Table 5. Similarly, if the slope coefficient value of the new coming candidate is greater than or equal to the right branch $\mu_{L1}+\sigma_{L1}$ of the variation interval, then the interpolation coefficient (C) will be equal to one, and the thickness of the right branch of the triangle system will be according to the formula $C=10-\frac{C+1}{2}\cdot 9+1$. Finally, if the slope coefficient value of the new candidate is greater than the left branch and less than the right branch $\mu_{L2}-\sigma_{L2}<New>\mu_{L1}+\sigma_{L1}$ of the variation interval within the distribution graph, then the interpolation coefficient will lie in between the variation interval, and the thickness of the left and right branches of the triangle system will be according to the formula shown in Table 5.

### 3.2. Classification of New Candidates

Now that we have the machine learning algorithms tuned up to the extracted knowledge from the two cohorts, we can demonstrate the efficiency and usefulness of the classification strategy by introducing new candidates. The self-organization of the cardiovascular systems of the two candidates during the load phase of the stress test is investigated by means of the developed machine learning algorithms.

**Figure 7:**
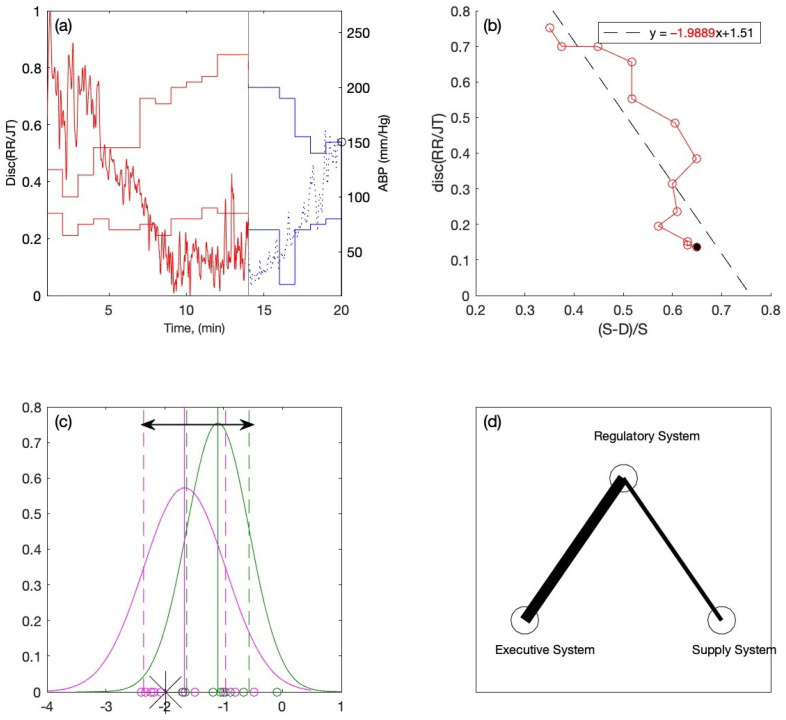
One case for an individual with (**a**) Discriminant RR/JT vs. time graph and ABP vs. time graph, (**b**) Linear regression graph and the slope coefficient (−1.9889) during the load phase, (**c**) Locating the individual within the variation interval of the Gaussian distribution graph, (**d**) Visualization of the individual inside the triangle system.

For the first candidate, the graph of the RR/JT algebraic relationship and the graph of the arterial blood pressure are overlapped in Figure 7a. It can be seen that the collapse of complexity occurs at the 14th minute of the stress test, notably during the load phase, and the individual is unable to continue the load. The blood pressure graph shows that the individual’s ABP appears to be more or less normal. Then, using the technique described in this study, the averaged algebraic relationships are used to construct the phase plane plots where the linear regression is performed over the load data (see Figure 7b). The equation of linear regression is depicted in Figure 7b where the slope coefficient value is shown in red. The linear regression slope coefficient value (−1.9889) is introduced to the Gaussian distribution plot in order to determine the person’s location within the variation interval (see Figure 7c). It can be observed that the new person’s slope coefficient fits inside the variation interval but is located to the far left. The resulting value of the indicator C is −0.5850. The thickness of the left branch of the triangle system becomes eight pixels, and the thickness of the right branch is three pixels (see Figure 7d).

The classification of slope coefficient of the new candidate into the category of the persons with an elevated ABP rate tells us that that his cardiovascular system is incapable of handling higher loads, and the pressure rises during the load to the limit point of the person’s regulatory system. It is worth noting that despite appearing to have normal ABP, the individual’s physiological response to the load was insufficient. The primary goal of this study is to develop such algorithms which are capable of highlighting such subtle differences.

Such situations may arise during antihypertensive therapy, when ABP is normal at the start of the load. However, as the load increases, ABP rises quickly, potentially leading to a slew of pathologic statements (heart ischemia accidents, stroke). The subtle difference of the individual in this case highlights the peculiarities of the cardiovascular system of a person while during the exercise—especially during the load—and this may help to generate an early warning for an individual.

**Figure 8:**
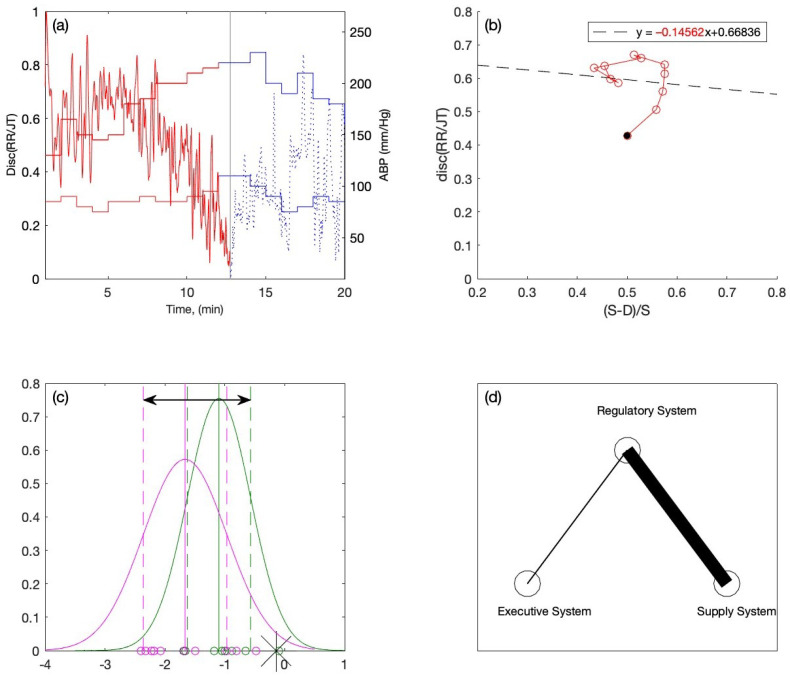
One case for an individual with (**a**) Discriminant RR/JT vs. time graph and ABP vs. time graph, (**b**) Linear regression graph and the slope coefficient (−0.14562) during the load phase, (**c**) Locating the individual within the variation interval of the Gaussian distribution graph, (**d**) Visualization of the individual inside the triangle system.

For the second individual, the RR/JT algebraic relationship and ABP data are presented in Figure 8a. According to the RR/JT relationship, the collapse of complexity occurs around the 13th minute of the activity during the load phase. The ABP graph values show that the individual has a high ABP (130/85 mmHg). The linear regression is performed on the load data, and the resulting equation of linear regression is depicted in Figure 8b. The slope coefficient value (−0.14562) is fitted into the variation interval of the Gaussian distribution plot (see Figure 8c). It appears that this individual falls outside the variation interval to the far right. The interpolation coefficient C is equal to 1, which results in the width of the right branch of the triangle system being ten pixels, and the width of the left branch of the triangle system being one pixel (see Figure 8d). Based on the results, the individual is classified to the category of persons with normal ABP (see Figure 8c). Despite the fact that the person appears to have high ABP from his ABP graph (see Figure 8b), his body’s response to load is considerably better compared to the previous person. The results of the classification explain his capacity to sustain rather high loads throughout the physical exercises.

It is also worth mentioning that we already have a dataset with a sample of individuals who had indications of the conditions known prior to the experiment—these conditions are the cohorts of people with normal ABP and the other cohort of people with increased ABP (Table 1 and Table 2). This classification allowed us to make clear distinctions between the groups of persons. Moreover, the introduction of the triangle system helps to determine the self-organization of their cardiovascular system during the stress test. This allows for the development of an early warning for persons who would not otherwise be identified as health-risky candidates. Since we are generating computer knowledge to categorize the new individual into this triangle system, based on our classification, we can refer to this computer-generated knowledge as a similar approach to the supervised learning technique.

The confusion matrix, the sensitivity, the specificity, the accuracy, and AUC-ROC curves could be used for the characterization of a classifier. Slope coefficients reconstructed from the data collected from 19 persons are used to build the model in this paper. However, the most important objective of this study is not the binary classification of the new candidate. The proposed triangular coordinate system (Figure 7d and Figure 8d) is not dichotomous. In other words, the thickness of the left and the right branches is proportional to the location of the candidate’s slope coefficient in the variation interval (Table 5). The introduced coordinate system helps to determine whether the new candidate’s cardiovascular response will be more marked by an increase in heart rate or an increase in blood pressure during a stress test. It may happen that the thickness of both branches would be equal, which would mark that the self-organization of the candidate’s cardiovascular system is located in between the two extremes. Therefore, techniques for the assessment of dichotomous classification between two distinct classes are not completely appropriate for such a problem.

## 4. Conclusions

A technique for assessing blood pressure regulation in a stress test is presented in this paper. Two categories of persons with normal and elevated blood pressure are exposed. Machine learning algorithms are used to build a set of rules and performance measures during a stress test. Heart rate, JT interval and blood pressure readings are observed during the loading and recovery phase of the exercise. This paper proves that subtle differences in the self-organization of the human cardiovascular system during the stress test are measurable and essential for detecting the individual performance of the candidate. The introduced coordinate system helps to determine whether the new candidate’s cardiovascular response will be more marked by an increase in heart rate or an increase in blood pressure during a stress test. The developed approach provides valuable information about the human cardiovascular system during the exercise.

This research study paves the way for identifying ABP dynamics during physical activity and avoiding cases where a sudden increase in ABP occurs or a person experiences hypertensive reactions and enters a hypertonic state. The establishment of such an early warning system based on machine learning algorithms can be especially beneficial for those who use antihypertensive medicines to control their blood pressure. Although blood pressure is kept under control, there may come a time when the cardiovascular system cannot handle the load and all self-regulating systems collapse, causing the blood pressure to rise and lead into an alarming health condition. This study leads to the identification of self-regulating system control mechanisms in the cardiovascular system and thereby could save lives.

## Figures and Tables

**Table 1:** Slope coefficients for individuals with normal ABP during the load and recovery. The goodness of the linear regression fit (for each individual slope coefficient) is measured by the Spearman’s rank correlation coefficient.

Persons with Normal ABP					
**No.**	**Names**	**Slope Coefficients during Load (W·ms),** ***M*** **1**	**Spearman’s Rank Correlation Coefficient during Load**	**Slope Coefficients during Recovery (W·ms),** ***M*** **2**	**Spearman’s Rank Correlation Coefficient during Recovery**
1	BACCHR	−1.6963	−0.8035	−0.32249	−0.8024
2	BARHEL	−0.65793	−0.9058	−0.012758	−0.0486
3	BROSOR	−1.6554	−0.9333	−0.30205	−0.4667
4	ENGBER	−1.0524	−0.8161	−0.19906	−0.8389
5	PETTHO	−1.0029	−0.8903	−0.90867	−0.9244
6	STEMAR	−0.097479	−0.2168	−0.25354	−0.8146
7	HEIRAL	−1.6539	−0.8908	−0.94723	−0.7538
8	FLOPET	−1.1824	−0.8389	−0.35497	−0.8024
9	SCHMAR	−0.88493	−0.9515	−0.16869	−0.8066

**Table 2:** Slope coefficients for persons with high ABP during the load and recovery. The goodness of the linear regression fit (for each individual slope coefficient) is measured by the Spearman’s rank correlation coefficient.

Persons with Normal ABP					
**No.**	**Names**	**Slope Coefficients during Load (W·ms),** ***M*** **3**	**Spearman’s Rank Correlation Coefficient during Load**	**Slope Coefficients during Recovery (W·ms),** ***M*** **4**	**Spearman’s Rank Correlation Coefficient during Recovery**
1	ADAWOL	−2.3278	−0.6960	−0.61569	−0.9119
2	BRODOR	−2.229	−0.7972	−0.66062	−0.9624
3	ILLBJO	−2.1799	−0.4745	−0.43314	−0.6869
4	NAUTHO	−1.4895	−0.9701	−0.48034	−0.9157
5	STETHO	−1.6843	−0.9066	−0.748	−0.9758
6	PFEAND	−2.3957	−0.8654	−0.152	−0.7833
7	NEUCHR	−0.48277	−0.6097	−0.13909	−0.8257
8	LINUWE	−0.97304	−0.4954	−0.14123	−0.7500
9	KRAHAR	−0.80256	−0.8742	−0.38448	−0.8074
10	KLITOR	−2.071	−0.9449	−0.63648	−0.9222

**Table 3:** Mean, standard deviation and significance values for the cohort of persons throughout the load and recovery process during the bicycle stress test.

	Mean (μ)	Std. Deviation (σ)	Confidence Interval	Sig. Level	Difference in Means	The Statistical Condition
Persons with normal ABP during the load	$\mu_{L1}=-1.0982$	$\sigma_{L1}=0.5287$	[−1.6269;−0.5695]	0.4636	$|\mu_{L2}-\mu_{L1}|=|-1.6636-\left(-1.0982\right)|=0.5654$	$|\mu_{L2}-\mu_{L1}|\geq min\left(\sigma_{L1},\sigma_{L2}\right)$ *Condition is satisfied*
Persons with high ABP during the load	$\mu_{L2}=-1.6636$	$\sigma_{L2}=0.6970$	[−2.3606;−0.9665]	0.1789		
Persons with normal ABP during the recovery	$\mu_{R1}=-0.3855$	$\sigma_{R1}=0.3239$	[−0.7094;−0.0616]	0.0190	$|\mu_{R2}-\;\mu_{R1}|=|-0.4391-\left(-0.3855\right)|=0.0536$	$|\mu_{R2}-\mu_{R1}|\geq min\left(\sigma_{R1},\sigma_{R2}\right)$ *Condition is not satisfied*
Persons with high ABP during the recovery	$\mu_{R1}=-0.4391$	$\sigma_{R1}=0.2311$	[−0.6702;−0.2080]	0.2339		

**Table 4:** Coordinates corresponding to the model of integral evaluation.

Coordinate	Corresponds to
−1, 0	E (the Executive System)
0, 1	R (the Regulatory System)
1, 0	S (the Supply System)

**Table 5:** Formalization of the three conditions for the slope coefficient of the new candidate entering into the system triangle.

	Condition 1	Condition 2	Condition 3
If	$New\leq \mu_{L2}-\sigma_{L2}$	$New\geq \mu_{L1}+\sigma_{L1}$	$\mu_{L2}-\sigma_{L2}<New>\mu_{L1}+\sigma_{L2}$
The interpolation coefficient	C=−1	C=1	C= in between the variation interval
The thickness of the left branch in pixels	$C=10-\frac{C+1}{2}\cdot 9$	1	$C=2\cdot \frac{New-(\mu_{L2}-\sigma_{L2})}{\left(\mu_{L1}+\sigma_{L1}\right)-\left(\mu_{L2}-\sigma_{L2}\right)}\cdot 9$
The thickness of the right branch in pixels	1	$C=\frac{C+1}{2}\cdot 9+1$	

## Data Availability

The data presented in this study are available on request from the corresponding author.

## Abbreviations

CNS	central nervous system
ABP	arterial blood pressure
HR	heart rate
MET	metabolic equivalent
SV	stroke volume
ABP (sys)	systolic arterial blood pressure
ABP (dis)	diastolic arterial blood pressure
JTa	amplitude of the JT interval
JTd	the duration of the JT interval
ECG	electrocardiogram
AHA	American Heart Association

## References

[1] Clermont G., Angus D. (2001). Towards understanding pathophysiology in critical care: The human body as a complex system. Yearbook of Intensive Care and Emergency Medicine 2001.

[2] Pocock G., Richards C.D., Richards D.A. (2013). Human Physiology.

[3] Žemaitytė M. (1997). Širdies Ritmo Autonominis Reguliavimas: Mechanizmai, Vertinimas, Klinikinė Reikšmė.

[4] Kevelaitis E.N.N., Menasché P. (1999). Coronary endothelial dysfunction of isolated hearts subjected to prolonged cold storage: Patterns and contributing factors. J. Heart Lung Transplant..

[5] Rowell L. (1997). Neural control of muscle blood flow: Importance during dynamic exercise. Clin. Exp. Pharmacol. Physiol..

[6] Hollander A., Bouman L. (1975). Cardiac acceleration in man elicited by a muscle-heart reflex. J. Appl. Physiol..

[7] Faria E., Faria I. (1998). Cardiorespiratory responses to exercises of equal relative intensity distributed between the upper and lower body. J. Sport. Sci..

[8] O’Sullivan S., Bell C. (2000). The effects of exercise and training on human cardiovascular reflex control. J. Auton. Nerv. Syst..

[9] Savin W.M., Davidson D.M., Haskell W.L. (1982). Autonomic contribution to heart rate recovery from exercise in humans. J. Appl. Physiol..

[10] Chapman J., Elliott P. (1988). Cardiovascular effects of static and dynamic exercise. Eur. J. Appl. Physiol. Occup. Physiol..

[11] Eriksen M.O., Waaler B.A., Walløe L., Wesche J. (1990). Dynamics and dimensions of cardiac output changes in humans at the onset and at the end of moderate rhythmic exercise. J. Physiol..

[12] Secher N.H., Clausen J.P., Klausen K., Noer I., Trap-Jensen J. (1977). Central and regional circulatory effects of adding arm exercise to leg exercise. Acta Physiol. Scand..

[13] Takahara K., Miura Y., Kouzuma R., Yasumasu T., Nakamura T., Nakashima Y. (1999). Physical training augments plasma catecholamines and natural killer cell activity. J. UOEH.

[14] O’Brien E., Pickering T., Asmar R., Myers M., Parati G., Staessen J., Mengden T., Imai Y., Waeber B., Palatini P. (2002). Working Group on Blood Pressure Monitoring of the European Society of Hypertension International Protocol for validation of blood pressure measuring devices in adults. Blood Press. Monit..

[15] Yamaguchi M., Shimizu M., Ino H., Okeie K., Yasuda T., Fujino N., Fujii H., Mabuchi T., Mabuchi H. (2000). Diagnostic usefulness of the post-exercise systolic blood pressure response for the detection of coronary artery disease in patients with diabetes mellitus. Jpn. Circ. J..

[16] Mansia G., De Backer G., Dominiczak A., Cifkova R., Fagard R., Germano G., Grassi G., Heagerty A.M., Kjeldsen S.E., Laurent S. (2007). 2007 Guidelines for the management of arterial hypertension: The Task Force for the Management of Arterial Hypertension of the European Society of Hypertension (ESH) and of the European Society of Cardiology (ESC). Eur. Heart J..

[17] Mancia G., Laurent S., Agabiti-Rosei E., Ambrosioni E., Burnier M., Caulfield M.J., Cifkova R., Clément D., Coca A., Dominiczak A. (2009). Reappraisal of European guidelines on hypertension management: A European Society of Hypertension Task Force document. Blood Press..

[18] McHam S.A., Marwick T.H., Pashkow F.J., Lauer M.S. (1999). Delayed systolic blood pressure recovery after graded exercise: An independent correlate of angiographic coronary disease. J. Am. Coll. Cardiol..

[19] Skirius J. Sportininkų širdies ir kraujagyslių sistemos funkcinės būklės tyrimas ir vertinimas. Proceedings of the Sporto Mokslo Dabartis ir Naujosios Idejos.

[20] Fletcher G.F., Balady G.J., Amsterdam E.A., Chaitman B., Eckel R., Fleg J., Froelicher V.F., Leon A.S., Piña I.L., Rodney R. (2001). Exercise standards for testing and training: A statement for healthcare professionals from the American Heart Association. Circulation.

[21] Bar-Yam Y. (1997). About Complex Systems. Reading.

[22] Fonseca C.G., Backhaus M., Bluemke D.A., Britten R.D., Chung J.D., Cowan B.R., Dinov I.D., Finn J.P., Hunter P.J., Kadish A.H. (2011). The Cardiac Atlas Project—An imaging database for computational modeling and statistical atlases of the heart. Bioinformatics.

[23] Bharti R., Khamparia A., Shabaz M., Dhiman G., Pande S., Singh P. (2021). Prediction of heart disease using a combination of machine learning and deep learning. Computational Intelligence and Neuroscience.

[24] Maurer M.S., Burkhoff D., Fried L.P., Gottdiener J., King D.L., Kitzman D.W. (2007). Ventricular structure and function in hypertensive participants with heart failure and a normal ejection fraction: The Cardiovascular Health Study. J. Am. Coll. Cardiol..

[25] Spyropoulos F., Vitali S.H., Touma M., Rose C.D., Petty C.R., Levy P., Kourembanas S., Christou H. (2020). Echocardiographic markers of pulmonary hemodynamics and right ventricular hypertrophy in rat models of pulmonary hypertension. Pulm. Circ..

[26] Rajput J.S., Sharma M., Tan R.S., Acharya U.R. (2020). Automated detection of severity of hypertension ECG signals using an optimal bi-orthogonal wavelet filter bank. Comput. Biol. Med..

[27] Soh D.C.K., Ng E., Jahmunah V., Oh S.L., Tan R.S., Acharya U. (2020). Automated diagnostic tool for hypertension using convolutional neural network. Comput. Biol. Med..

[28] Jain P., Gajbhiye P., Tripathy R., Acharya U.R. (2020). A two-stage deep CNN architecture for the classification of low-risk and high-risk hypertension classes using multi-lead ECG signals. Inform. Med. Unlocked.

[29] Parmar K.S., Kumar A., Kalita U. (2022). ECG signal based automated hypertension detection using fourier decomposition method and cosine modulated filter banks. Biomed. Signal Process. Control.

[30] Li H., Deng J., Feng P., Pu C., Arachchige D.D., Cheng Q. (2021). Short-Ter, Nacelle Orientation Forecasting Using Bilinear Transformation and ICEEMDAN Framework. Front. Energy Res.

[31] Li H., Deng J., Yuan S., Feng P., Arachchige D.D. (2021). Monitoring and Identifying Wind Turbine Generation Bearing Faults Using Deep Belief Network and EWMA Control Charts. Front. Energy Res..

[32] Batzel J.J., Kappel F., Schneditz D., Tran H.T. (2007). Cardiovascular and Respiratory Systems: Modeling, Analysis, and Control.

[33] Šiaučiūnaitė V., Ragulskis M., Vainoras A., Dabiri B., Kaniusas E. (2021). Visualization of complex processes in cardiovascular system during electrical auricular vagus nerve stimulation. Diagnostics.

[34] Stock M., Ryan M. (1996). Oxygen consumption calculated from the Fick equation has limited utility. Crit. Care Med..

[35] Delong C., Sharma S. (2019). Physiology, Peripheral Vascular Resistance.

[36] Armstrong R.B., Warren G.L., Warren J.A. (1991). Mechanisms of exercise-induced muscle fibre injury. Sport. Med..

[37] Rowel L. (1993). Circulatory responses to upright posture. Human Cardiovascular Control: Reflex Control During Orthostasis.

[38] Gargasas L., Vainoras A., Schwela H., Jaruševičius G., Ruseckas R., Miškinis V. JT interval changes during bicycle ergometry. Proceedings of the Kardiologia Polska II Miedzynarodowy Kongres Polskiego Towarzystwa Kardiologieznego.

[39] Shaffer F., McCraty R., Zerr C.L. (2014). A healthy heart is not a metronome: An integrative review of the heart’s anatomy and heart rate variability. Front. Psychol..

[40] McCraty R., Atkinson M., Tomasino D., Bradley R.T. (2009). The coherent heart heart-brain interactions, psychophysiological coherence, and the emergence of system-wide order. Integral Rev. Transdiscipl. Transcult. J. New Thought Res. Prax..

[41] Segerstrom S., Nes L. (2007). Heart rate variability reflects self-regulatory strength, effort, and fatigue. Psychol. Sci..

[42] Woods K. (2000). QT Dispersion in Ischaemic Heart Disease.

[43] Roukema G., Singh J.P., Meijs M., Carvalho C., Hart G. (1998). Effect of exercise-induced ischemia on QT interval dispersion. Am. Heart J..

[44] Yoshimura M., Yasue H., Ogawa H. (2001). Pathophysiological significance and clinical application of ANP and BNP in patients with heart failure. Can. J. Physiol. Pharmacol..

[45] Vainoras A., Gargasas L., Ruseckas R., Miškinis V., Jurkonienė R. Computerised exercise electrocardiogram analysis system “Kaunas-Load”. Proceedings of the 24th International Congress on Electrocardiology and 38th International Symposium on Vectorcardiography: Abstracts Book.

[46] Gargasas L., Vainoras A., Ruseckas R., Jurkoniene R., Jurkonis V., Miskinis V. A new software for ECG monitoring system. Proceedings of the 6th Nordic Conference on eHealth and Telemedicine.

[47] Ziaukas P., Alabdulgader A., Vainoras A., Navickas Z., Ragulskis M. (2017). New approach for visualization of relationships between RR and JT intervals. PLoS ONE.

[48] Saunoriene L., Siauciunaite V., Vainoras A., Bertasiute V., Navickas Z., Ragulskis M. (2019). The characterization of the transit through the anaerobic threshold based on relationships between RR and QRS cardiac intervals. PLoS ONE.

[49] Laio F. (2004). Cramer–von Mises and Anderson-Darling goodness of fit tests for extreme value distributions with unknown parameters. Water Resour. Res..

